# Does Folcisteine (NATCA) Play a Role in Facilitating Seed Germination, Root Development, and Elevating Root AsA-GSH Cycle Efficiency Under Combined Copper–Cadmium Stress in Maize?

**DOI:** 10.3390/ijms262211220

**Published:** 2025-11-20

**Authors:** Ling Dong, Meng Zhao, Kangbo Hou, Jingwen Wei, Ziwen Liu, Runze Wang, Yu Zhou, Wanrong Gu

**Affiliations:** College of Agriculture, Northeast Agricultural University, Harbin 150030, China; dongling@neau.edu.cn (L.D.); zmeng1116@163.com (M.Z.); s220301018@neau.edu.cn (K.H.); s2503022090@neau.edu.cn (J.W.); s2503022063@neau.edu.cn (Z.L.); s2503012109@neau.edu.cn (R.W.)

**Keywords:** maize, folcisteine, seed germination, root development, AsA-GSH cycle, copper–cadmium stress

## Abstract

Copper (Cu) and cadmium (Cd) are common co-occurring environmental pollutants inducing combined stress, which severely harms maize growth. Previous studies have confirmed the involvement of the ascorbate–glutathione (AsA-GSH) cycle in heavy metal stress tolerance, but the regulatory effect of NATCA on this cycle under Cu-Cd combined stress—especially during maize seed germination and root development—remains unelucidated. Exogenous folcisteine (NATCA, 3-acetylthiazolidine-4-carboxylic acid) can enhance plant tolerance to abiotic stress. This study investigated the role of NATCA, a novel plant growth regulator with antioxidant potential, in alleviating Cu-Cd combined stress in maize. Two maize (*Zea mays* L.) varieties—Jiuyuan 15 (Cu/Cd-tolerant) and Longfuyu 6 (Cu/Cd-intolerant)—were exposed to combined stress (80 mg·L^−1^ CuSO_4_ + 100 mg·L^−1^ CdCl_2_) with/without 20 mg·L^−1^ NATCA. Germination and hydroponic experiments were conducted to investigate NATCA’s effects on seed germination, growth, root traits, photosynthetic characteristics, reactive oxygen species (ROS) metabolism, AsA-GSH cycle (ascorbate–glutathione cycle), and endogenous hormones under stress. The results showed that combined Cu/Cd stress inhibited seed germination (reduced vigor, rate, index), while NATCA significantly reversed these declines, increased tolerance index, lowered relative damage rate, and improved seed activity—with more pronounced effects on Longfuyu 6. Stress stunted seedling growth (reduced dry/fresh weight, water content; increased water deficit), whereas NATCA promoted growth (taller plants, less leaf chlorosis, more fibrous roots), enhanced dry matter accumulation, and improved water metabolism. Stress impaired root morphology (shorter length, smaller surface area/volume, fewer tips) and absorption capacity; NATCA improved root traits, stress tolerance, and vitality. Stress weakened ROS scavenging, but NATCA elevated antioxidant enzyme activity and non-enzymatic antioxidant content, strengthened AsA-GSH cycle-mediated ROS clearance, mitigated stress damage, and maintained intracellular ROS balance in maize seedling root. These findings not only reveal a new regulatory role of NATCA in enhancing heavy metal stress tolerance via the AsA-GSH cycle but also provide a potential eco-friendly strategy for improving maize production in heavy metal-contaminated soils.

## 1. Introduction

Soil heavy metal pollution, a complex and insidious environmental issue, presents a formidable challenge precisely because it defies direct assessment and intuitive perception by humans. Unlike more overt forms of pollution, such as visible waste dumps or oil slicks on water surfaces, heavy metal contaminants within the soil matrix remain concealed from our immediate senses. This inability to readily detect or gauge their presence through straightforward observational means has led to soil heavy metal pollution emerging as an elusive hidden danger that lurks silently beneath our feet, yet has the potential to wreak havoc on various ecological and human systems [1]. Characterized by concealment, latency, cumulativeness, and irreversibility, heavy metal contamination of soil is often overlooked despite its severe detrimental effects [2]. The damage inflicted on soil ecosystems by heavy metals is irreversible, and remediation of contaminated soil remains technically challenging and economically costly. Atmospheric deposition constitutes a primary source of regional soil heavy metal pollution [3]. Additionally, extensive discharge of industrial wastewater into farmland as irrigation water contributes significantly to such pollution [4,5]; notably, excessive application of phosphorus fertilizers can elevate soil cadmium (Cd) content. Concurrently, Cd pollution arising from atmospheric deposition and agricultural irrigation is on the rise [6]. The global concern over heavy metal pollution in agricultural soils is exemplified by China’s 2014 survey, which revealed exceedance rates of 16.1% for soil and 19.4% for arable land, with Cu and Cd notably impairing crop yield, quality, and human health via the food chain [7,8,9]. A 2018 report revealed moderate heavy metal pollution in southern Songnen Plain, Heilongjiang, with Cu and Cd being predominant, evidenced by a concentration of 42.8 mg·kg^−1^ in a carex scoparia swamp and 0.77% of samples exhibiting moderate Cd pollution, respectively [10,11]. Thus, investigating methods to mitigate heavy metal harm to crops and their underlying response mechanisms holds significant importance.

The phytotoxicity of both essential Cu and non-essential Cd manifests in suppressed seed germination and growth through the disruption of metabolic processes, including limited starch fixation, nutrient supply, and decreased proteolytic enzyme activity [12]. The suppressive effect of Cu and Cd on seed germination potential, rate, and index is primarily mediated by disrupting metabolic homeostasis—affecting soluble sugar and trace element metabolism—and by inhibiting the activities of α-amylase and acid phosphatase [13]. Phytotoxicity assays revealed that Cu^2+^ and Cd stress significantly inhibit seed germination and early seedling growth in white clover and wheat, adversely affecting key parameters such as germination rate, potential, and root morphology [14,15]. Research has found that plants treated with Cd^2+^ exhibit symptoms such as short and brown roots, reduced lateral roots, slow stem growth, yellowing, curling, and spotted leaves, short stature, and yellowing of leaves [16]. By inhibiting the activity of starch-hydrolyzing enzymes like α-amylase, high-concentration Cd treatment disrupts the mobilization of energy reserves, leading to insufficient metabolic processes for seed germination [17,18,19,20]. Cu and Cd can bind to the catalytic sites of proteolytic enzymes, thereby disrupting protein hydrolysis in seeds. This impairment leads to insufficient nitrogen supply to the embryo, ultimately hindering seed germination [21]. Additionally, Cu and Cd treatments disrupt the physiological balance of seedlings, resulting in reduced carbon and nitrogen metabolism, decreased chlorophyll content, damaged cell membrane structure, and subsequent cell fluid leakage [22]. As Cu and Cd accumulate in plants, the extent of damage intensifies, manifesting as stunted growth, abnormal leaf coloration, and overall inhibited seedling development [23].

Root system morphology, critical for Cu and Cd uptake and xylem transport following initial metal perception, plays a definitive role in regulating the metal’s accumulation within the plant [24,25,26]. Exposure to Cu and Cd significantly inhibits seedling root growth, leading to reduced length, number, and volume, as well as yellowing and curling, which consequently impairs water and nutrient uptake [27]. Previous studies indicate that Cu^2+^ concentration is positively correlated with the root inhibition rate in grasses, demonstrating a clear dose-dependent inhibition of growth [28]. Most plant species exhibit higher concentrations of Cu and Cd in roots than in stems, with limited translocation of these metals to the xylem [29]. The subcellular accumulation of Cd in roots, primarily within the symplast and cell walls, exhibits a dynamic distribution that is highly dependent on cultivation conditions [30]. Over 81% of Cd in rice roots is compartmentalized within the soluble fraction and cell wall, owing to the presence of Cd-complexing ligands such as organic acids, amino acids, and phytochelatins that facilitate its accumulation [31]. The Cu and Cd ions adsorbed on maize roots, primarily precipitated in the epidermis and outer layers, are partitioned into exchangeable, complex-bound, and precipitated fractions, which in turn dictate their specific biological impacts [32]. Most Cu^2+^ and Cd^2+^ bind to the cell wall, leading to cross-linking of pectin in the middle layer and inhibiting cell elongation and growth, thereby suppressing root length [33,34,35,36,37]. Functioning as a key regulatory mechanism, the AsA-GSH cycle enhances plant tolerance by mitigating oxidative damage induced by diverse abiotic stresses [38]. Research indicates that enhancing the activity of key enzymes and the pool sizes of AsA and GSH within the AsA-GSH cycle effectively reduces reactive oxygen species (ROS) levels, thereby boosting plant stress tolerance [39]. In plant cells, ascorbate peroxidase (APX) scavenges H_2_O_2_, generating monodehydroascorbic acid (MDHA); MDHA further converts to dehydroascorbic acid (DHA). Monodehydroascorbate reductase (MDHAR) facilitates ascorbic acid (AsA) regeneration and MDHA scavenging, while dehydroascorbate reductase (DHAR) primarily reacts with DHA to regenerate AsA [40,41,42]. Glutathione (GSH) can reduce oxidized glutathione (GSSG) back to GSH; AsA reduces free metal ions and maintains antioxidant enzyme activity, and GSH acts as an antioxidant [43,44,45,46,47,48]. Previous studies on plant leaves have found that stress initially induces phytotoxicity in leaves, characterized by a suppression of key antioxidant enzymes (APX, GR, MDHAR, DHAR), yet subsequently triggers an adaptive response marked by proline accumulation and the recovery of APX and GR activities [49,50].

Folcisteine (NATCA), also known as 3-acetylthiazolidine-4-carboxylic acid or N-acetylthioproline, is a derivative of organic amino acids. The molecular formula of NATCA is C_6_H_9_NO_3_S, with a molecular weight of 175.2056. Folcisteine is an intermediate drug used to treat myocardial ischemia. NATCA is used in agriculture as a plant growth regulator. Alleviating heavy metal-induced damage to plants via exogenous substances has become a current research focus. Specifically, the application of certain exogenous substances can alter the speciation of heavy metals, mitigate their inhibitory effects on plant growth, stimulate the expression of stress-resistant genes, reduce their toxicity by regulating bioavailability, and ultimately promote plant growth [51]. Using maize varieties of differential heavy metal sensitivity, this study aimed to investigate how exogenous NATCA modulates seed germination, root growth, and the root AsA-GSH cycle under Cu-Cd co-stress, offering new methodologies and a theoretical basis for improving maize tolerance.

## 2. Results

### 2.1. Evaluation of Germination and Copper–Cadmium Tolerance Parameters During Stress

As shown in Table 1, NATCA treatment enhanced the germination potential, rate, and index of both maize varieties compared to CK, whereas CuCd stress caused a decline in these parameters. After NATCA treatment of Jiuyuan 15 and Longfuyu 6, compared with CK, the germination vigor of the two varieties increased by 5.79% and 25.93%, the germination rate increased by 1.39% and 2.81%, and the germination index increased by 17.23% and 18.23%, respectively. After CuCd treatment, the germination potential of Jiuyuan 15 and Longfuyu 6 decreased by 23.91% and 61.72%, respectively, compared to the CK, the germination rate decreased by 20.83% and 23.24%, respectively, and the germination index decreased by 31.20% and 46.85%, respectively. The decline in germination vigor, germination rate, and germination index of Longfuyu 6 is higher than that of Jiuyuan 15, indicating that Jiuyuan 15 has a higher ability to resist CuCd than Longfuyu 6. Compared with CuCd, after NATCA + CuCd treatment, the germination potential of Jiuyuan 15 and Longfuyu increased by 25.93% and 25.71%, the germination rate increased by 2.81% and 20.17%, the germination index increased by 18.23% and 31.15%, the relative damage rate decreased by 75.52% and 76.22%, and the CuCd tolerance index increased by 31.19% and 69.85%, respectively. The CuCd tolerance index of Longfuyu 6 treated with NATCA + CuCd was 69.87% higher than that of Jiuyuan 15, indicating that NATCA has a more significant effect on the germination of Longfuyu 6 seeds (Table 1).

### 2.2. Growth Phenotype of Maize Seedlings

**Figure 1:**
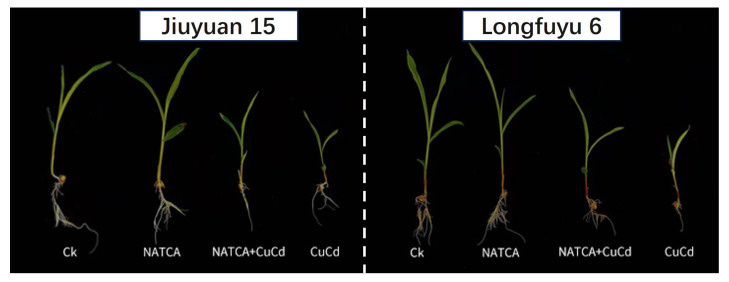
Ameliorative effect of NATCA on the toxicity of copper–cadmium combined stress in maize seedlings.

From Figure 1, it can be clearly observed that the growth of maize seedlings treated with CuCd is inhibited, resulting in stunted growth, yellowing and wilting of leaves, white stripes at the edge of leaf tips, slow and short root growth, less rooting, and yellowing and darkening of roots. Among the two varieties, Longfuyu 6 showed more significant damage after being subjected to CuCd stress. Compared with CuCd treatment, NATCA + CuCd treatment reduced the area of leaf chlorosis and improved root growth in both varieties, with an increase in the number and length of fibrous roots. Compared with CK, NATCA treatment resulted in the best growth of maize seedlings, with high plant height, wide leaves, and well-developed root systems (Figure 1).

### 2.3. Dry and Fresh Weight, Moisture Content, and Natural Saturation Deficit

As indicated in Table 2, NATCA-treated maize seedlings exhibited the highest fresh weight, dry weight, and moisture content, along with the lowest natural saturation deficit, whereas the CuCd-treated group showed the opposite trend across all parameters. The effects of different treatments on the fresh weight, dry weight, and natural saturation deficit of Jiuyuan 15 and Longfuyu 6 maize seedlings are summarized in Table 2. Compared to the CK group, NATCA treatment alone increased fresh and dry weights in both varieties, whereas CuCd stress significantly reduced fresh weight (by 43.25% and 36.83%, respectively) and dry weight, and markedly increased the natural saturation deficit (by over 120%). The application of NATCA + CuCd mitigated the stress-induced damage, leading to increases in fresh and dry weights and a reduction in the natural saturation deficit by more than 54% compared to the CuCd treatment alone (Table 2).

### 2.4. Root Characteristic Parameters

As summarized in Table 3, CuCd stress significantly inhibited maize root growth, as evidenced by reductions in root length, diameter, surface area, volume, and tip number, whereas NATCA treatment promoted these morphological parameters. Root length increased under NATCA (by up to 71.79% in Jiuyuan 15) but decreased under CuCd stress; NATCA + CuCd treatment resulted in a net increase compared to CK (by 56.60% and 17.66%). Average diameter saw a moderate increase with NATCA and a decrease with CuCd, but was recovered and even promoted beyond CK levels with NATCA + CuCd, especially in Longfuyu 6 (+16.83% vs. CuCd). Root surface area and volume followed a similar pattern, with NATCA + CuCd treatment effectively reversing the losses caused by CuCd stress. Total root tip number was the most responsive parameter, dramatically enhanced by NATCA (by 246.94% in Jiuyuan 15), severely reduced by CuCd, and remarkably restored by NATCA + CuCd, with increases exceeding 159% over the CuCd treatment (Table 3).

### 2.5. Activities of Key Enzymes in the Root Ascorbate–Glutathione (AsA-GSH) Cycle in Maize Root

**Figure 2:**
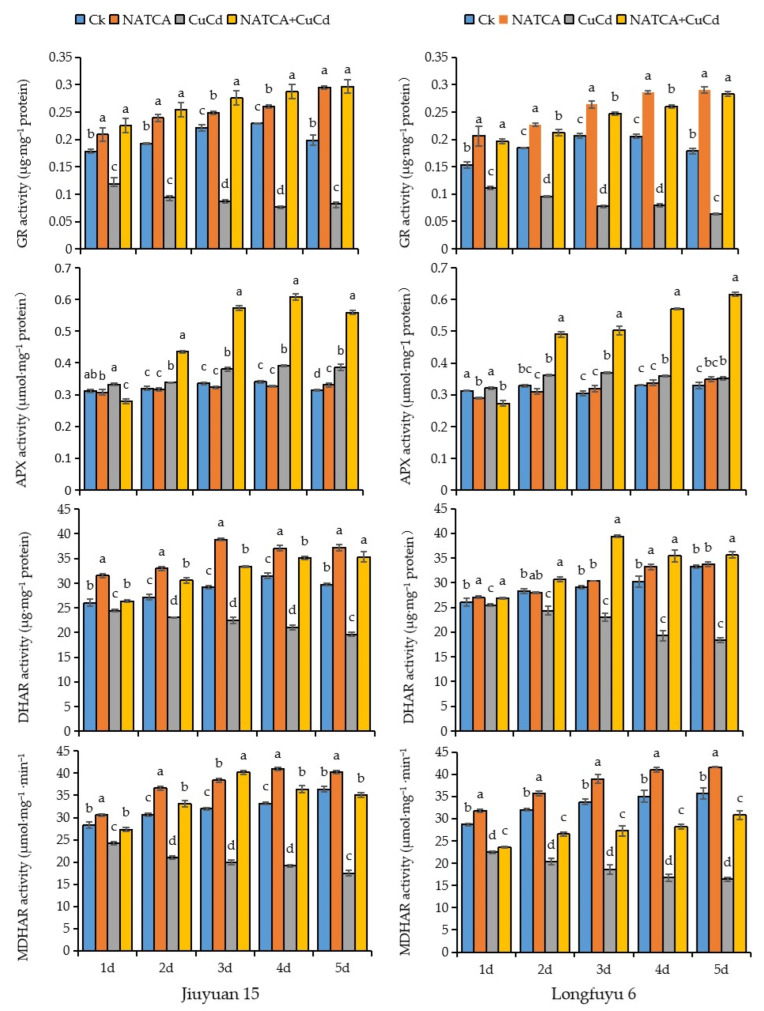
Influence of NATCA on the operation of the AsA-GSH cycle in roots under copper–cadmium combined stress. Values are mean ± SD (*n* = 3). Significant differences (*p* < 0.05) as determined by Duncan’s multiple range test are indicated by different letters within a column for the following treatments: NATCA, which represents 1/2 Hoagland nutrient solution containing 20 mg·L^−1^ NATCA; CuCd, which represents 1/2 Hoagland nutrient solution containing 80 mg·L^−1^ CdCl_2_ and 100 mg·L^−1^ CuSO_4_; and NATCA + CuCd, which represents 1/2 Hoagland nutrient solution containing 20 mg·L^−1^ NATCA, 80 mg·L^−1^ CdCl_2_, and 100 mg·L^−1^ CuSO_4_.

As the primary site perceiving CuCd stress, maize roots exhibited altered AsA-GSH cycle enzyme activities (Figure 2), while APX activity increased under stress compared to CK, GR, DHAR, and MDHAR decreased, and these suppressed activities were restored by NATCA supplementation. As shown in Figure 2, CuCd stress induced a complex time-dependent response in the root antioxidant enzyme activities of both maize varieties. While CuCd treatment initially increased APX activity but markedly decreased GR, DHAR, and MDHAR activities over 1–5 days compared to CK, the application of NATCA + CuCd not only further enhanced APX activity but also dramatically reversed the suppression of the other three enzymes. The restorative effect of NATCA was particularly strong on GR activity, which saw increases of over 160% in Jiuyuan 15 and 120% in Longfuyu 6 by day 5 compared to the CuCd treatment (Figure 2).

### 2.6. Contents of Ascorbic Acid (AsA) and Glutathione (GSH) in Maize Root

**Figure 3:**
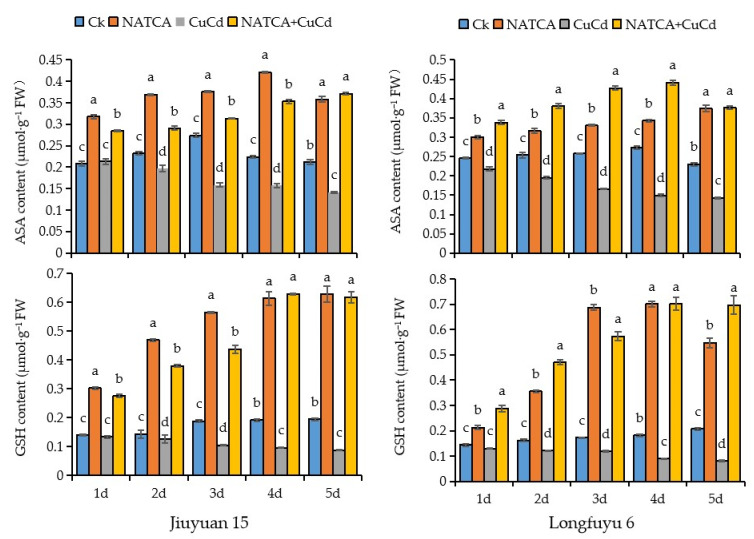
Effects of NATCA on the content of AsA and GSH in roots under combined copper—cadmium stress. Significant differences (*p* < 0.05) as determined by Duncan’s multiple range test are indicated by different letters within a column for the following treatments: NATCA, which represents 1/2 Hoagland nutrient solution containing 20 mg·L^−1^ NATCA; CuCd, which represents 1/2 Hoagland nutrient solution containing 80 mg·L^−1^ CdCl_2_ and 100 mg·L^−1^ CuSO_4_; and NATCA + CuCd, which represents 1/2 Hoagland nutrient solution containing 20 mg·L^−1^ NATCA, 80 mg·L^−1^ CdCl_2_, and 100 mg·L^−1^ CuSO_4_.

AsA and GSH, as crucial reducing substances in plants, play a key role in preventing membrane lipid peroxidation. As shown in Figure 3, compared with the CK treatment, CuCd stress led to a significant time-dependent decrease in the contents of AsA and GSH in the roots of both maize varieties. However, the addition of NATCA (NATCA + CuCd treatment) effectively reversed this trend, resulting in a substantial increase in the contents of AsA and GSH compared to the CuCd treatment alone. The increase in GSH content was particularly remarkable, with an elevation ranging from 106% to 760% over 1–5 days, indicating that NATCA alleviates oxidative damage by significantly enhancing the ASA-GSH cycle. Overall, compared with the CuCd treatment, the application of NATCA + CuCd increased the contents of AsA and GSH in maize roots. For instance, compared with the CuCd treatment, the AsA content in the roots of Jiuyuan 15 seedlings treated with NATCA + CuCd for 1–5 days increased by 33.49%, 47.38%, 97.9%, 125.05%, and 162.5%, respectively. Similarly, the AsA content in Longfuyu 6 increased by 55.05%, 94.89%, 156.72%, 194.87%, and 162.8%, respectively. Meanwhile, the GSH content in Jiuyuan 15 increased by 106.26%, 201.03%, 317.5%, 547.76%, and 596.59%, respectively, while that in Longfuyu 6 increased by 121.28%, 285.52%, 382.35%, 686.22%, and 760.9%, respectively.

In contrast, compared with the CK treatment, CuCd stress resulted in a decrease in the contents of AsA and GSH in maize roots. Specifically, in Jiuyuan 15, the AsA content showed no significant difference on the first day after CuCd treatment compared with CK, but decreased by 15.09%, 42.22%, 29.81%, and 33.23% on days 2–5, respectively. In Longfuyu 6, the AsA content decreased by 11.38%, 23.1%, 35.53%, 45.18%, and 37.68% over 1–5 days, respectively. The GSH content in Jiuyuan 15 decreased by 4.76%, 12.09%, 44.23%, 49.3%, and 54.61% over 1–5 days, respectively, while that in Longfuyu 6 decreased by 9.51%, 24.54%, 30.81%, 50.92%, and 60.87%, respectively. These results demonstrate that, compared with CK, CuCd stress caused a significant time-dependent reduction in the contents of AsA and GSH in the roots of both maize varieties, with the maximum decrease in GSH content exceeding 60%. In contrast, the addition of NATCA (NATCA + CuCd treatment) markedly elevated the levels of these two antioxidants. Particularly in the later stages of treatment, the GSH content showed a dramatic increase, by up to 547.76% to 760.9%, indicating that NATCA effectively reversed the inhibitory effect induced by CuCd stress (Figure 3).

## 3. Discussion

The germination process, which marks a plant’s first encounter with heavy metal stress, is characterized by parameters such as vigor, rate, and index that collectively reflect seed vitality [52]. CuCd stress reduces seed activity and inhibits germination, as evidenced by decreased germination potential, rate, and index alongside increased relative damage rate in maize—consistent with prior findings that combined CuCd stress lowers these parameters in five wolf grass species [53,54]. Seed germination in Phoenix tail cockscomb was subject to concentration-dependent inhibition by Cu^2+^ [55,56]. This experimental study found that the length of the embryonic root decreased, and embryonic roots exhibited wilting after CuCd treatment. Previous studies have demonstrated that high concentrations of Cu^2+^ markedly suppress the germination and early seedling growth of adzuki beans [57]. In the germination experiment, it was also found that the embryonic roots of seeds treated with CuCd turned yellow and wilted, possibly due to the embryonic roots breaking through the seed coat first, resulting in the accumulation of more CuCd ions and causing greater damage to the embryonic roots. This study demonstrated that NATCA treatment significantly enhanced the germination potential, rate, and index of maize seedlings, suggesting its efficacy in breaking seed dormancy and improving overall seed vigor. According to literature analysis, it is possible that NATCA reversed the synthesis of ABA, promoted the production of ethylene, and disrupted the primary and secondary dormancy of seeds [58,59]. NATCA, a plant growth regulator, enhances seed germination and alleviates dormancy by modulating key enzymes and proteins through signal transduction and hormonal crosstalk. This study further confirms that NATCA treatment significantly reduces seed damage and improves CuCd tolerance in maize. This finding demonstrates that NATCA mitigates the phytotoxic impact of Cu-Cd co-exposure on germination, thereby enhancing seed tolerance throughout the germination process [59].

After being stimulated by Cu and Cd, maize will activate self-protection mechanisms to reduce or avoid the entry of Cu and Cd into the plant body as much as possible in order to better adapt to the environment. At the cellular level, the cell wall is a structure that was exposed to Cu and Cd stress earlier [60]. Due to the anionic nature of D-galacturonic acid in cell wall pectins, which exhibit a strong exchange capacity for divalent and trivalent cations such as Cu^2+^ and Cd^2+^, these metal ions are effectively immobilized within the cell wall, thereby preventing their entry into the cell [61]. The tolerance and defense response of maize to Cd^2+^ stress is associated with significant alterations in cell wall polysaccharide components and their ratios to lignin and cellulose, suggesting a key role for cell wall remodeling in metal sequestration [62]. Beyond functioning as a primary barrier for toxic metal accumulation, the cell wall also dynamically adjusts its structure in response to cadmium stress as an adaptive mechanism. A key adaptive change is the increase in low-methylated pectin, which enhances the binding of divalent/trivalent metal ions, thereby boosting the cell wall’s accumulation capacity and reducing their translocation to the protoplast [63,64,65,66]. Maize roots employ the secretion of low-molecular-weight organic acids (LMWOAs) as a strategy to mitigate the phytotoxic effects induced by heavy metal stress. The low molecular weight organic acids secreted by plant roots can alter the migration of cadmium ions in soil after forming soluble complexes [67]. The LMWOA secreted by plant roots, such as oxalic acid, malic acid, succinic acid, and citric acid, helps to form soluble complexes and chelates, and alters the mobility of heavy metals in the soil rhizosphere [68]. The immobilization of heavy metals in the soil significantly reduces the translocation of Cd, Ni, and Zn to various plant parts [69]. Metals can chelate with ligands outside of cells, such as through the chelation of organic acids flowing from roots, as well as metallothioneins (MT) and phytochelatins (PC).

Cu is an essential trace element for plants, while Cd is non-essential; both can induce plant growth stress when their environmental concentrations exceed a certain threshold. Cu-Cd combined stress inhibits maize seedling growth (e.g., stunted growth, wilting, shorter height, yellow leaves) and exerts similar effects on tomatoes, such as shortened, slender stems and yellow leaves [70]. Research shows Cd stress significantly reduces the plant height, stem thickness, fresh weight, and dry weight of thin-skinned cantaloupe [71]. This study indicates that the dry and fresh weight decreases after CuCd treatment, which inhibits the accumulation of dry matter in maize seedlings. This conclusion is consistent with previous research results. Higher Cd concentrations reduce wheat seedling height, slow leaf extension, and significantly decrease fresh weight, dry weight, and water content [72]. Research has shown that as the concentration of copper and cadmium increases, the plant height and individual weight of Ogi tend to decrease [73]. This study showed that NATCA application enhanced maize growth, resulting in thicker stems, wider and longer leaves, and increased dry and fresh weight. These findings indicate that NATCA can promote maize seedling growth and boost biomass. The study also showed that applying NATCA to maize seedlings under CuCd stress improved leaf yellowing, restored normal growth, and increased plant height, dry weight, and fresh weight. These results indicate that NATCA can alleviate the inhibitory effect of CuCd stress on maize seedling growth. The water status in plants is one of the important indicators for studying plant stress physiology. Natural saturation deficiency reflects the internal water shortage in plants. This study indicates that under CuCd stress, plant water content decreases while natural saturation deficit increases, suggesting maize seedlings suffer from water shortage. Most abiotic stresses (e.g., salt, drought, heavy metal stress) disrupt plant water balance, which is linked to the regulation of aquaporin subtype expression and activity [74]. Plant water absorption is closely related to water channel proteins, and cadmium accumulation can cause oxidative damage to lipids and proteins and reduce antioxidant enzyme activity, thereby affecting plant water status [75].

To survive under heavy metal stress, plants evolve morphological structures that avoid or tolerate heavy metals, with one key form being limiting root absorption of heavy metals [76]. This study shows that CuCd stress damages maize seedling roots—causing curling, yellowing, shortening, and fewer lateral roots—and research has found that such combined stress hinders root growth, with higher Cu and Cd content in roots than in aboveground parts. Under combined Cu-Cd stress, root accumulation of Cu and Cd is higher than under single Cu or Cd stress [77]. Cd stress can cause damage to root growth, inhibit root elongation, and significantly reduce root growth [78]. The root is the maize organ that directly contacts soil; as soil Cu and Cd concentrations gradually increase, various root indicators show a downward trend, and Cu-Cd stress can damage chromosomes and nucleoli—with stronger copper toxicity to nucleoli leading to a more potent inhibitory effect on root growth [79,80]. The inhibition of root elongation by Cu and Cd may be due to the interference of heavy metals on cell division, including inducing chromosomal aberrations and mitotic abnormalities [81]. The phenotypic changes in roots treated with Cu may be related to auxin. Under Cu stress, auxin activity is higher in the meristematic and elongation zones of roots. Excessive auxin accumulation in the meristematic and elongation zones may reduce cell division and elongation, causing primary root growth arrest [82]. Cd stress reduces root growth, followed by a significant decrease in cell viability, an increase in PAL and POD activity and H_2_O_2_ levels. This may affect the production and further polymerization of phenylpropane derivatives, as well as increase the production of lignin and its H and S units. Therefore, cadmium-induced root growth inhibition may be due to the excessive production of mono lignin, which polymerizes to form lignin, hardening the cell wall and limiting plant growth [83]. This study showed that NATCA treatment increased maize seedlings’ root length, average diameter, surface area, volume, and total root tip number, indicating NATCA can promote root growth. Previous studies found that 2-(5-bromo-2-hydroxy-phenyl)-thiazolidine-4-carboxylic acid (BHTC) induces plant resistance to bacterial, oomycetous, and fungal pathogens, and low-dose BHTC promotes Arabidopsis root growth [84]. This study found that NATCA alleviates CuCd-induced root damage, restoring curled and yellow roots to normal white and firm states, and increasing lateral root number and root length. This study also found that in CuCd treatment, the growth direction of roots changed to horizontal and closely adhered to the hydroponic floating plate above the water surface as much as possible. After adding NATCA, the root system resumed downward growth, the degree of decay also decreased, and the number of primary roots increased.

Copper and cadmium stress indirectly induce oxidative stress in plants by generating ROS. The level of AsA GSH cycle is usually positively correlated with the expression of related enzyme genes, that is, high expression of related enzyme genes promotes the progression of AsA GSH cycle, increases its circulation level, and better maintains the homeostasis of ROS in plant cells. The ascorbic acid glutathione cycle is the main pathway for antioxidant defense, mainly detoxifying ROS in plant cells [85]. This study suggests that CuCd stress reduces root GR activity—possibly because DHAR, which participates in converting DHA to AsA, is involved in GR generation, leading to consistent changes in root GR and DHAR activities—and that the antioxidant system, beyond its detoxification function, may also be a sensitive target of Cd toxicity in plants. Low Cd levels increase APX activity in maize leaves—an early adaptive response of APX to Cd stress that may serve as a prospective biomarker for soil Cd in maize seedlings, and high AsA and GSH contents can protect cells from Cu- and Cd-induced oxidative damage [86]. This study indicates that under CuCd stress, the levels of AsA and GSH in maize seedlings decrease. Previous studies found that under Cu stress, AsA and GSH contents in wheat roots decreased compared to the control group, which may be due to tissue-absorbed copper ions reaching a threshold, leading to AsA and GSH accumulation in tissues [87]. In plants, antioxidant reactions mainly rely on the utilization and recycling of GSH and AsA—GSH not only has a primary antioxidant function but also serves as a substrate for regenerating other essential antioxidants (e.g., AsA)—and in the AsA-GSH cycle, the two oxidized metabolites (GSSG and DHA) are recycled by NADPH/GR and GSH/DHAR, respectively [88,89]. In this study, the roots were more affected by oxidative stress caused by CuCd, and NATCA had the most significant effect on the roots. In this study, only the physiological responses of maize to CuCd stress after NATCA treatment were observed, such as alleviated oxidative damage and improved growth indicators. It is speculated that this may involve the regulation of hormones such as ABA or ethylene, but this mechanism has not been directly verified through experiments such as determining endogenous hormone contents or analyzing the expression of genes related to hormone synthesis/signaling transduction. Therefore, the specific association between NATCA and plant hormone pathways still needs to be further clarified in subsequent studies through targeted determination and intervention experiments. One limitation of this study is the lack of data on copper and cadmium accumulation. Although this study focused on internal physiological responses, future research should determine if NATCA affects metal uptake beyond its observed role in mitigating oxidative stress, as this will provide a more complete understanding of the interaction between this compound and heavy metal stress in maize seedlings.

## 4. Materials and Methods

### 4.1. Experimental Materials

Based on preliminary experiments, Jiuyuan 15 (tolerant to CuCd stress) and Longfuyu 6 (intolerant to CuCd stress) were selected as materials, with seeds provided by Gansu Zhongyu Seed Industry Co., Ltd. (Zhangye, China) and Jilin Rongsheng Seed Industry Co., Ltd. (Changchun, China), respectively. Half leaf extract (NATCA, Folcisteine) was purchased from Zhengzhou Zhengshi Chemical Products Co., Ltd. (Zhengzhou, China), CAS number: 5025-82-1, molecular formula C_6_H_9_NO_3_S, molecular weight 175.2056. Copper stress was treated with copper sulfate pentahydrate, molecular formula CuSO_4_.5H_2_O, molecular weight: 249.685, CAS number: 7758-99-8, provided by Tianjin Third Chemical Plant Co., Ltd. (Tianjin, China). Cadmium stress was treated with anhydrous cadmium chloride, molecular formula CdCl_2_, molecular weight: 183.32, CAS number: 10108-64-2, provided by Shenyang Xinguang Chemical Plant (Shenyang, China).

### 4.2. Growth Condition of Seed Germination

Select 50 healthy and plump seeds from each of the two varieties, disinfect them with 10% sodium hypochlorite for 10 min, and clean them thoroughly. Put it into a culture dish with filter paper and divide it into four treatments: (1) distilled water (CK); (2) 20 mg·L^−1^ NATCA; (3) 80 mg·L^−1^ CdCl_2_ + 100 mg·L^−1^ CuSO_4_ (CuCd); (4) 20 mg·L^−1^ NATCA + 80 mg·L^−1^ CdCl_2_ + 100 mg·L^−1^ CuSO_4_ (NATCA + CuCd), setting up 3 replicates. Place the seeds in a constant temperature incubator for dark cultivation at 26 °C and 96% humidity. To ensure concentration, change the filter paper once a day. The number of germinated seeds was counted at fixed daily intervals, with germination defined as the stage when the lengths of the radicle and plumule reached half the length of the seed. CuCd tolerance index (%) = (mean of a certain growth index in the stress treatment group ÷ mean of the same growth index in the control group) × 100.

### 4.3. Growth Condition of Seedling Hydroponic Cultivation

Healthy and plump seeds were selected for this experiment, disinfected with 10% sodium hypochlorite solution, washed and soaked for 10 h, evenly spread into germination boxes, and transferred to a constant temperature incubator (26 ± 1 °C). After the seeds germinate and the embryo grows to 1.5–2 cm, select germinated seeds with consistent growth and plant them in a non-light culture pot containing 20 L of 1/2 Hoagland nutrient solution (pH = 6.0 ± 0.1). Change the nutrient solution once in 3D, ventilate the air pump at regular intervals (45 min·h^−1^), expose to light for 12 h, and maintain a temperature of 28 ± 1 °C. When the seedlings grow to three leaves and one center, select seedlings with consistent growth for treatment. Each group has 60 seedlings, divided into four groups.

The experiment comprised four treatments: the control (CK) with 1/2 Hoagland nutrient solution; the NATCA treatment with 20 mg·L^−1^ NATCA added to the solution [90]; the copper–cadmium stress (CuCd) treatment with 80 mg·L^−1^ CdCl_2_ and 100 mg·L^−1^ CuSO_4_ [91,92]; and the combined NATCA + CuCd treatment with 20 mg·L^−1^ NATCA, 80 mg·L^−1^ CdCl_2_, and 100 mg·L^−1^ CuSO_4_. Add 80 mg·L^−1^ CdCl_2_ and 100 mg·L^−1^ CuSO_4_ to Hoagland nutrient solution in maize three-leaf stage. The initial pH was adjusted to 6.0 ± 0.1, and measurements taken daily confirmed that the pH remained within this range (5.9–6.1) throughout the experimental period. The seedlings that require CuCd treatment should be added to the copper–cadmium treatment solution twice, with an interval of 12 h, to provide a buffer time for the seedlings. Seedlings that require NATCA treatment should be treated with copper–cadmium compound for 12 h before adding NATCA. Adjust pH once a day, with a photoperiod of 12/12 (day/night), temperature of (28 ± 1) °C/(25 ± 1) °C day/night, light intensity of around 400 μmol·m^−2^·s^−1^, and relative humidity of 60% to 70%. The 12th hour after adding various treatments is taken as the 1st day of sampling.

### 4.4. Determination of Seedling Growth Phenotypic

On the 5th day of treatment, select three maize seedlings with consistent growth in each treatment, clean them thoroughly, absorb the water with filter paper, and weigh their fresh weight. Kill at 105 °C for 30 min in an oven, and dry at 80 °C until constant weight. Weigh the dry weight separately and calculate the moisture content.

On the 5th day of treatment, three maize seedlings with consistent growth were selected for each treatment, and their leaf parts were taken. After weighing its fresh weight, soak the leaves in distilled water for 6 h, remove them, absorb the water with filter paper, and weigh them. Soak in distilled water for another 30 min, remove and dry the leaves, weigh them until they reach saturation weight to obtain the saturated fresh weight, then perform withering and drying to measure the dry weight, and calculate the water saturation deficit.

### 4.5. Determination of Root Characteristic Parameters

On the 5th day after treatment, the roots of maize seedlings were cut and washed. Three plants were taken from each treatment and washed with distilled water. The root morphology was scanned and analyzed using EPSON1680 root scanner (Winrhizo2005a root analysis software).

### 4.6. Determination of GR Activities

Take maize seedling roots (0.5 g) with consistent growth from each treatment at regular intervals every day for 1–5 days after treatment, and separate the roots. Glutathione reductase (GR) can catalyze the reduction in oxidized glutathione (GSSG) by nicotinamide adenine dinucleotide phosphate (NADPH) to regenerate reduced glutathione (GSH), while continuously consuming NADPH. The activity of GR can be determined by measuring the rate of decrease in absorbance at 340 nm due to the consumption of NADPH (with a molar extinction coefficient of 6.22 mM^−1^ cm^−1^). The reaction mixture (1 mL) contains 100 mM sodium phosphate buffer (pH 7.8), 0.5 mM GSSG, 50 mL of extract, and 0.1 mM NADPH. One unit of GR activity is defined as the amount of enzyme that oxidizes 1 nmol of NADPH per minute per milligram of protein under the conditions of 25 °C and pH 8.0. Cut each sample into pieces, add PBS (pH = 7.4), grind to a homogenate at 2–8 °C, and leave for 20 min (3000 r/min) to obtain the supernatant. The GR activity was measured using the glutathione reductase activity detection kit provided by Nanjing Jiancheng Bioengineering Institute (Nanjing, China, http://www.njjcbio.com/) and assessed on 5 January 2022. Measure the absorbance using a spectrophotometer (340 nm) within 15 min after adding the stop solution.

### 4.7. Determination of APX Activities

Take maize seedling roots with consistent growth from each treatment at regular intervals every day for 1–5 days after treatment. The activity of APX was determined by monitoring the oxidation rate of ascorbate at 290 nm (with a molar extinction coefficient of 2.8 mM^−1^ cm^−1^). The reaction mixture (3 mL) contained 1.5 mL of 0.1 M potassium phosphate buffer (pH 6.8), 0.5 mL of 6 mM ascorbate, 0.5 mL of 12 mM H_2_O_2_, and 0.5 mL of enzyme extract.

### 4.8. Determination of DHAR Activities

The preliminary preparation work is the same as the steps for GR activity determination. DHAR activity was detected using the dehydroascorbate reductase (DHAR) activity detection kit provided by Nanjing Jiancheng Bioengineering Institute (http://www.njjcbio.com/) and assessed on 5 January 2022, and the absorbance was measured using a spectrophotometer. DHAR activity was calculated by measuring the change in absorbance at 340 nm over 4 min due to DHA oxidation (with a molar extinction coefficient of 14 mM^−1^ cm^−1^). The reaction mixture comprised 90 mM K-phosphate buffer (pH 7.0), 1 mM EDTA, 5.0 mM GSH, and enzyme extract. The reaction was initiated by the addition of 0.2 mM dehydroascorbic acid.

### 4.9. Determination of MDHAR Activities

The preliminary preparation work is the same as the steps for GR activity determination. The MDHAR activity was detected using the MDHAR activity detection kit from Nanjing Jiancheng Bioengineering Research Institute (http://www.njjcbio.com/) and assessed on 5 January 2022, and the absorbance was measured using a spectrophotometer. MDHAR activity was calculated by measuring the change in absorbance at 340 nm over 4 min due to NADH oxidation (with a molar extinction coefficient of 6.2 mM^−1^ cm^−1^). The reaction mixture (1 mL) contained 90 mM K-phosphate buffer (pH 7.0), 0.0125% Triton X-100, 0.2 mM NADH, 2.5 mM ascorbate, 0.25 units of ascorbate oxidase, and enzyme extract. One unit of MDHAR activity was defined as the amount of enzyme that oxidizes 1 μmol of NADH per minute per milligram of protein.

### 4.10. Determination of AsA Contents

Take maize seedling roots with consistent growth from each treatment at regular intervals every day for 1–5 days after treatment. Grind 0.5 g of the sample with 5% hydrochloric acid, centrifuge 15,000× *g* for 20 min, and take the supernatant as the crude extract. Then add PBS and water to the supernatant (supernatant: 150 Mm PBS: water = 1:1:1). Vortex the mixture and let it stand at room temperature for 1 min. Add 10% TCA (Trichloroacetic Acid), 45% H_3_PO_4_, and 3% FeCl_3_ for vortexing. Maintain at 37 °C for 1 h and measure using a spectrophotometer.

### 4.11. Determination of GSH Contents

Prepare the crude extract as shown in the AsA content determination, take the supernatant to measure the total content of GSH and GSSG, add the crude enzyme solution to the reaction solution, and then perform colorimetric analysis. Measure the content of GSSG again, add the crude enzyme solution diluted 50 times to the pyrimidine water bath for colorimetric analysis, and subtract the two to obtain the GSH content.

### 4.12. Data Analysis

The data were expressed by the measured mean value, analyzed by SPSS19.0 (IBM SPSS Statistics, 2010), and compared by Duncan’s new complex difference method (α = 0.05), and origin 8 is used for drawing.

## 5. Conclusions

Exogenous NATCA enhances maize seed germination and alleviates root growth inhibition by improving root traits under combined Cu/Cd stress. Additionally, NATCA regulates root development via modulating the AsA-GSH cycle, thus protecting root cell structure.

## Figures and Tables

**Table 1:** Alleviating effects of NATCA on seed germination under copper–cadmium combined stress by modulating physiological responses in maize.

Variety	Treatment	Germination Vigor (%)	Germination Rate (%)	Germination Index	Relative Damage Degree	Tolerance Index
Jiuyuan 15	Ck	92.00 ± 0.12 a	96.00 ± 0.16 a	26.44 ± 0.12 b		
	NATCA	97.33 ± 0.06 a	97.33 ± 0.67 a	31.00 ± 5.67 a		
	CuCd	70.00 ± 0.02 c	76.00 ± 0.02 b	18.19 ± 2.33 d	0.20 ± 0.34	0.69 ± 0.01
	NATCA + CuCd	88.00 ± 0.23 b	91.33 ± 0.31 a	23.86 ± 2.31 c	0.05 ± 0.36	0.90 ± 0.02
Longfuyu 6	Ck	54.00 ± 0.24 b	94.67 ± 1.76 ab	15.78 ± 4.18 b		
	NATCA	68.00 ± 0.02 a	97.33 ± 0.91 a	18.66 ± 9.01 a		
	CuCd	20.67 ± 0.18 d	72.67 ± 0.55 c	8.39 ± 5.46 c	0.23 ± 3.23	0.53 ± 0.04
	NATCA + CuCd	35.33 ± 0.03 c	90.67 ± 0.13 b	14.27 ± 2.88 b	0.05 ± 0.02	0.90 ± 0.02

Notes: Data are expressed as mean ± standard deviation, where NATCA denotes 1/2 Hoagland nutrient solution containing 20 mg·L^−1^ NATCA; CuCd denotes 1/2 Hoagland nutrient solution containing 80 mg·L^−1^ CdCl_2_ and 100 mg·L^−1^ CuSO_4_; and NATCA + CuCd denotes 1/2 Hoagland nutrient solution containing 20 mg·L^−1^ NATCA, 80 mg·L^−1^ CdCl_2_, and 100 mg·L^−1^ CuSO_4_, with different letters in the same column indicating a significant difference at the 5% level.

**Table 2:** Response of dry matter and water content to NATCA under copper–cadmium combined stress on the fifth day in maize seedlings.

Cultivars	Treatment	Fresh Weight (g·plant^−1^)	Dry Weight (g·plant^−1^)	Water Content (%)	Natural Saturation Deficit (%)
Jiuyuan 15	CK	6.15 ± 0.06 b	0.51 ± 0.01 b	91.78 ± 0.09 a	2.38 ± 0.01 d
	NATCA	7.17 ± 0.15 a	0.56 ± 0.01 a	92.24 ± 0.01 a	3.95 ± 0.01 c
	CuCd	3.49 ± 0.03 d	0.50 ± 0.04 b	85.76 ± 0.05 b	28.34 ± 0.01 a
	NATCA + CuCd	4.36 ± 0.11 c	0.57 ± 0.01 a	86.91 ± 0.01 b	7.70 ± 0.02 b
Longfuyu 6	CK	5.24 ± 0.03 b	0.61 ± 0.04 b	87.48 ± 0.05 b	7.28 ± 0.02 c
	NATCA	6.04 ± 0.04 a	0.66 ± 0.07 a	90.00 ± 0.03 a	12.71 ± 0.06 b
	CuCd	3.31 ± 0.01 d	0.46 ± 0.01 d	84.03 ± 0.02 c	26.24 ± 0.05 a
	NATCA + CuCd	3.66 ± 0.01 c	0.53 ± 0.04 c	87.39 ± 0.07 b	13.19 ± 0.06 b

Notes: Data are expressed as mean ± standard deviation, where NATCA denotes 1/2 Hoagland nutrient solution containing 20 mg·L^−1^ NATCA; CuCd denotes 1/2 Hoagland nutrient solution containing 80 mg·L^−1^ CdCl_2_ and 100 mg·L^−1^ CuSO_4_; and NATCA + CuCd denotes 1/2 Hoagland nutrient solution containing 20 mg·L^−1^ NATCA, 80 mg·L^−1^ CdCl_2_, and 100 mg·L^−1^ CuSO_4_, with different letters in the same column indicating a significant difference at the 5% level.

**Table 3:** Alleviating effect of NATCA on copper–cadmium stress by modifying root architectural traits at day 5 in maize.

Cultivars	Treatment	Length (cm)	Average Diameter (cm)	Surface Area (cm^2^)	Volume (cm^3^)	Root Tips
Jiuyuan 15	Ck	8.69 ± 0.53 c	1.09 ± 0.06 a	3.03 ± 0.04 c	0.08 ± 0.05 ab	170.00 ± 6.08 a
	NATCA	14.93 ± 0.90 a	1.11 ± 0.03 a	4.60 ± 0.02 a	0.11 ± 0.02 a	49.00 ± 8.50 c
	CuCd	7.28 ± 0.28 c	0.98 ± 0.07 a	2.49 ± 0.18 d	0.07 ± 0.02 b	28.67 ± 4.48 c
	NATCA + CuCd	11.41 ± 0.54 b	1.05 ± 0.03 a	3.78 ± 0.28 b	0.10 ± 0.08 ab	78.00 ± 6.08 b
Longfuyu 6	Ck	16.07 ± 1.02 ab	1.00 ± 0.09 bc	5.46 ± 0.29 a	0.14 ± 0.05 b	214.00 ± 18.33 a
	NATCA	17.33 ± 0.93 a	1.16 ± 0.04 a	5.61 ± 0.23 a	0.16 ± 0.04 a	101.00 ± 9.29 b
	CuCd	11.61 ± 0.61 c	0.95 ± 0.08 c	4.24 ± 0.03 b	0.12 ± 0.04 b	37.00 ± 2.31 c
	NATCA + CuCd	13.66 ± 0.45 bc	1.11 ± 0.02 ab	4.70 ± 0.17 b	0.13 ± 0.04 b	96.00 ± 4.36 b

Notes: Data are expressed as mean ± standard deviation, where NATCA denotes 1/2 Hoagland nutrient solution containing 20 mg·L^−1^ NATCA; CuCd denotes 1/2 Hoagland nutrient solution containing 80 mg·L^−1^ CdCl_2_ and 100 mg·L^−1^ CuSO_4_; and NATCA + CuCd denotes 1/2 Hoagland nutrient solution containing 20 mg·L^−1^ NATCA, 80 mg·L^−1^ CdCl_2_, and 100 mg·L^−1^ CuSO_4_, with different letters in the same column indicating a significant difference at the 5% level.

## Data Availability

The original contributions presented in this study are included in the article. Further inquiries can be directed to the corresponding authors.
